# Supplementary material for: Content-rich biological network constructed by mining PubMed abstracts
Source: BMC Bioinformatics. 2004 Oct 8;5:147. doi: 10.1186/1471-2105-5-147 (PMC528731; doi:10.1186/1471-2105-5-147)
Supplement: Additional File 2 — The original results of the above study (non-essential files are deleted to keep the file size under the limit set by BMC bioinformatics). [file 1471-2105-5-147-S2.bz2 › chilibotAdditionalFile2/dip30/172ID9388212E587/html/IL6ST_TYK2.html]

 


 **IL6ST** and **TYK2** 
  
Found 27 abstracts in PubMed, retrieved 27.  
 

 What does Google say? 
 PDF only 
| .edu only 

---

**Interactive relationship** (e.g. stimulation, inhibition, etc)

**Neutral relationship**- Bcr gp130  [ **IL6ST** ]  is capable of interacting with JAK1, JAK2, and  **TYK2** .  Ref: 9388212 J Biol Chem, 1997
- The signal transduction of gp130  [ **IL6ST** ]  involves the Janus tyrosine kinases JAK JAK1, JAK2 and  **Tyk2**  and then the downstream effectors comprising the signal transducer and activator of transcription 3 STAT3 and mitogen activated protein kinase MAPK pathways.  Ref: 10929036 Br J Haematol, 2000
- Regarding the mechanisms whereby ku and gp130  [ **IL6ST** ]  signaling are coupled in PBMC, results from co immunoprecipitation experiments have shown that ku in the cytoplasm of PBMC from young, but not from elderly subjects, is associated with Tyk 2  [ **TYK2** ] , a kinase involved in signal transduction events after gp130  [ **IL6ST** ]  triggering by IL 6 type cytokines.  Ref: 11772519 Exp Gerontol< MedlineTA>Exp Gerontol, 2000
- Moreover, transactivation of gp130  [ **IL6ST** ]  was critically dependent upon Janus kinase activation by the initiating receptor and correlated with rapid and sustained Janus kinase 1 and tyrosine kinase Tyk 2  [ **TYK2** ]  tyrosine phosphorylation.  Ref: 12646637 J Immunol, 2003

**Non-interactive relationship** (e.g. studied together, co-existance, homology, etc.)

- The Janus kinases Jak1, Jak2,  **Tyk2**  and gp130  [ **IL6ST** ]  show intermediate half lives.  Ref: 10491180 Eur J Biochem, 1999
- Mechanical stretch induced rapid phosphorylation of JAK1, JAK2,  **Tyk2** , STAT1, STAT3, and glycoprotein 130  [ **IL6ST** ]  as early as 2 minutes and peaked at 5 to 15 minutes.  Ref: 10347087 Circ Res, 1999
- PTPepsilonC expression resulted in lower levels of IL 6 induced tyrosine phosphorylation of Jak1,  **Tyk2** , gp130  [ **IL6ST** ] , and Stat3 compared with parent cells.  Ref: 10859312 J Biol Chem, 2000
- Moreover, the present results indicate that after gp130  [ **IL6ST** ]  signaling both Tyk 2  [ **TYK2** ]  and ku 80 are phosphorylated, suggesting their activation by K 7 D 6.  Ref: 11772519 Exp Gerontol< MedlineTA>Exp Gerontol, 2000
- The key components involved are the signal transducing receptor subunit gp130  [ **IL6ST** ] , the Janus kinases Jak1, Jak2 and  **Tyk2** , STAT1 and STAT3 of the family of signal transducers and activators of transcription, the protein tyrosine phosphatase SHP2 and the suppressors of cytokine signalling SOCS1, SOCS2 and SOCS3.  Ref: 10491180 Eur J Biochem, 1999
- IL 6 induced transient tyrosine phosphorylation of the IL 6 receptor signal transducing subunit gp130  [ **IL6ST** ] , the gp130  [ **IL6ST** ]  associated protein tyrosine kinases Jak1, Jak2, and  **Tyk2** , the phosphotyrosine phosphatase PTP1D Syp, the adaptor protein Shc and the mitogen activated protein kinase Erk2, and accumulation of GTP bound p21ras.  Ref: 8978300 Blood, 1997
- LIF 10 3 U mL induced rapid tyrosine phosphorylation of gp130  [ **IL6ST** ] , JAK1, JAK2, STAT1, and STAT3 but not  **Tyk2**  or STAT2.  Ref: 9351438 Circ Res, 1997
